# Cutaneous clear cell/signet-ring cell squamous cell carcinoma arising in the right thigh of a patient with type 2 diabetes: combined morphologic, immunohistochemical, and etiologic analysis

**DOI:** 10.1186/s13000-016-0487-1

**Published:** 2016-04-11

**Authors:** Nong-Rong Wang, Meng-Meng Wang, Lv Zhou, Ze-Lin Liu, Nan-Ping Chen, Jin-Ping Hu, Yan-Juan Deng, Xiao-Qing Qi, Xiao-Feng Huang, Yue Su, Si-Yao Zhang, Fei Tong, Yu Zhang, Qi Lu, Zi-Yu Zhu, Huan Deng

**Affiliations:** Molecular Medicine and Genetics Center, The Fourth Affiliated Hospital of Nanchang University, Nanchang, China; Department of Pathology, The Fourth Affiliated Hospital of Nanchang University, 133 South Guangchang Road, Nanchang, 330003 China; Department of Endocrinology, The Fourth Affiliated Hospital of Nanchang University, Nanchang, China; Medical College, Nanchang University, Nanchang, China; Renmin Institute of Forensic Medicine, Nanchang, China

**Keywords:** Clear cell, Signet-ring cell, HPV, Diabetes mellitus, Squamous cell carcinoma

## Abstract

**Background:**

The clear cell/signet-ring cell variant of cutaneous squamous cell carcinoma (cSCC) is extremely rare. Its carcinogenesis has consistently been linked to ultraviolet radiation and HPV in the literature. However, there is little definite information about the contribution of diabetes mellitus (DM) to cSCC.

**Case presentation:**

A 78-year-old Chinese woman with type 2 DM presented with a mushroom-like lump in her right thigh. Histological findings revealed that the lesion was mainly composed of clear cells and signet-ring cells. The septa of vacuoles in cytoplasm displayed positivity for periodic acid schiff (PAS) and cytokeratins such as AE1/AE3, CK5/6, CK14, and CK19. Malignant cells did not express CK7, CK8, CK18, CK20, p16, p53, or c-erbB-2, and the Ki-67 index was less than 5 %. We further explored the etiology of clear cell/signet-ring cell cSCC using human papillomavirus (HPV) type-specific PCR and genotyping and confirmed that the patient was not infected with HPV. Nucleus positivity for p63 indicated the involvement of the p53 family in the lesion. Meanwhile, the expression of fibroblast growth factor receptor-2 (FGFR2), a downstream effector of p63, was upregulated in tumor cells.

**Conclusions:**

This study provides the first report on the clear cell/signet-ring cell variant of cSCC found in the right thigh of a patient with type 2 DM. Metabolic imbalance in addition to conventional pathogens such as UV and HPV may contribute to the development of the lesion via p63/FGFR2 axis.

## Background

The clear cell/signet-ring cell morphology has historically been considered to be a feature of mucin-producing adenocarcinoma in the literature. Primary cutaneous squamous cell carcinomas (cSCC) with clear cell/signet-ring cell morphology are very rare and have always been reported in sun-exposed regions of the body, such as the head and neck [1]. The phrase “clear cell” refers to cells characterized by hydropic cytoplasm, and “signet-ring cell” is used to describe cells characterized by eccentric nuclei that are located near the cellular border by large cytoplasmic vacuoles [2]. Among cutaneous carcinomas, the existence of the clear cell/signet-ring cell provides a basis for differential diagnosis, ruling out a variety of adnexal tumors [3, 4].

The exact etiology of the clear cell/signet-ring cell variant of cSCC is largely unknown. A main pathogen for cutaneous SCC is exposure to ultraviolet (UV) radiation, since more than 80 % of these tumors appear on sun-exposed parts of body [5]. Human papillomaviruses (HPVs) from the β genus are suspected to act as a co-carcinogen with UV. An early region of the HPV genome encodes five or six early non-structural proteins, including E1, E2, E4, E5, E6 and E7 [6]. The High-risk HPV proteins E6 and E7 are able to promote malignant transformation of squamous cells by degrading the tumor suppressor p53 [7]. The risk for developing cSCC appears to be 65–250 times higher in immunosuppressed patients, especially organ transplant recipients (OTR) [8].

Although the precise molecular mechanisms are still the subject of controversy, several lines of evidence suggest a close relationship between diabetes mellitus (DM) and many tumors including those of the pancreas, liver, and breast [9–11]. Recent studies have provided substantial evidence that DM may contribute to the pathogenesis of cSCC. Primary cSCC presenting as chronic diabetic foot ulcers has been proposed as a complication of DM [12–15]. More recently, a elaborate study demonstrated that oral SCC in DM patients was associated with poorer prognoses compared to oral SCC in non-DM patients [16]. Furthermore, oral administration of metformin, a traditional anti-DM drug, can significantly improve the overall survival of DM patients with head and neck cSCC [17]. In this study, we report the first case of cutaneous SCC with clear cell/signet-ring cell morphology found in the right thigh of a woman with type 2 DM. Immunohistochemistry and HPV type-sepcific PCR and genotyping were employed to explore the possibly underlying mechanisms.

## Case presentation

**Fig. 1:**
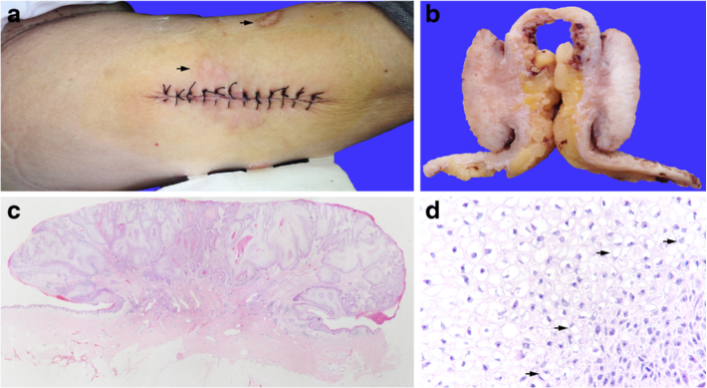
Clinical and histological findings of clear cell/signet ring cell cSCC. A mushroom-like lesion in right thigh was resected under local anesthesia (**a**, **b**). Scars caused by chronic ulcer were observed adjacent to the lesion (**a**, *black arrow*). Clear cell/signet ring cell assembled thick trabecula and solid nest (**c**, original magnification × 20). Scattered signet ring cells were observed (**d**, *black arrow*, original magnification × 400)

A 78-year-old Chinese woman, gravida 2, para 2, with menopause at age 51, presented to the Department of Endocrinology with a 2-week history of 5 × 3 × 1.5 cm mushroom-like lump in her right thigh (Fig. 1a, b). According to the medical history, the patient was a housewife and confirmed that she was not exposed to excessively professional, accidental, or medical UV radiation. She had a 30-year history of type 2 DM and her HbA1c was 8.4 % (68mMol/mol) upon admission. There were several scars caused by chronic ulcers adjacent to the lesion (Fig. 1a). She denied any history of neoplasms.

The lesion was resected under local anesthesia. The sample was fixed in 4 % buffered formalin and embedded in paraffin using conventional techniques. Serial tissue sections were studied using hematoxylin and eosin staining, histochemistry, immunohistochemistry (Table 1), and HPV type-specific PCR and genotyping. Blind evaluation of all results was performed by two independent pathologists.

**Table 1:** Summary of Primary Antibodies Used for Immunohistochemistry

Antibody	Supplier	Dilution
Cytokeratin	DAKO	1:100
Cytokeratin 5/6	DAKO	1:100
Cytokeratin 7	DAKO	1:100
Cytokeratin 8	DAKO	1:150
Cytokeratin 14	DAKO	1:150
Cytokeratin 18	DAKO	1:100
Cytokeratin 19	DAKO	1:100
Cytokeratin 20	DAKO	1:100
c-erbB-2	DAKO	1:100
P16	abcam	1:150
P53	abcam	1:100
P63	abcam	1:100
FGFR2	abcam	1:150

### Histopathology

Light microscopy examination showed that the lesion was mainly composed of malignant clear cells and signet-ring cells arranged in thick trabeculae or solid nests (Fig. 1c). The clear cells contained prominent vacuoles which were sharply demarcated and appeared empty. Each of the signet-ring cells contained a large cytoplasmic vacuole and an eccentric nucleus (Fig. 1d). Atypical mitotic figures were plentiful.

### Immuno- and histochemistry

**Fig. 2:**
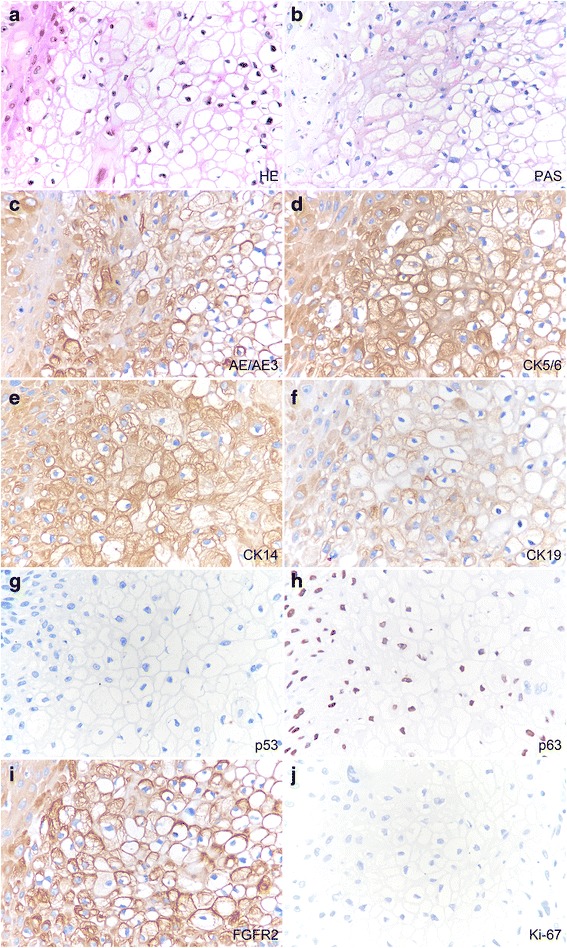
Histochemical and immunohistochemical profiles of clear cell/signet-ring cell SCC. The lesion was composed of diffused clear cells/signet cells (**a**, original magnification × 400). The septa of clear cells/signet-ring cells in serial sections were positive for PAS (**b**, original magnification × 400). They also expressed cytokeratin AE1/AE3, CK5/6, CK14, and CK19 (**c**–**f**, original magnification × 400). The lesion did not exhibit mutations of p53 (**g**, original magnification × 400). P63 positivity was detected in the nuclei of malignant cells (**h**, original magnification × 400). Clear cells/signet cells expressed strong-diffuse reactivity for FGFR2, a downstream effector of p63 (**i**, original magnification × 400). Less than 5 % of cancer cells expressed Ki-67 (**j**, original magnification × 400)

Periodic-acid Schiff (PAS), Alcian blue (AB), and mucicarmin techniques were employed to explore the nature of the observed clear cell/signet ring cell structures. The septa rather than the vacuoles showed positivity for PAS indicating the existence of glycogen (Fig. 2a, b). However, neither septa nor vacuoles expressed AB or mucicarmine, suggesting that neither mucin nor mucopolysaccharides existed (data not shown). In line with the above results, the septa expressed cytokeratin AE1/AE3, CK5/6, CK14, and CK19 (Fig. 2c-f). CK5/6 is a high molecular weight cytokeratin wihic is usually up-regulated in neoplasms of epithelial origin, including cSCC. Although malignant cells did not express mutant p53, they displayed strong and diffuse positivity for p63, another member of the p53 family playing an important role in normal epithelial development and differentiation (Fig. 2g, h). We also found the increased expression of fibroblast growth factor receptor-2 (FGFR2), a downstream effector of p63 (Fig. 2i). Nucleus immunoreactivity for Ki-67 is a hallmark of high cell proliferation. In the sections, less than 5 % of cancer cells expressed Ki-67 (Fig. 2j). Malignant cells exhibited negativity for CK7, CK8, CK18, CK20, P16, and c-erbB-2 (data not shown).

### HPV type-specific PCR and genotyping

**Fig. 3:**
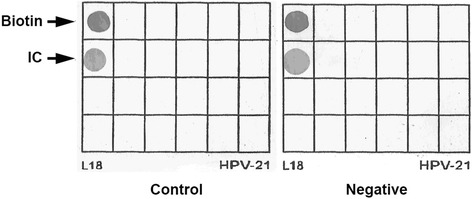
The results of HPV type-specific PCR and genotyping indicated that the patient was negative for infection with HPV

DNA was extracted from FFPE tissue sections and purified using the TIANamp FFPE DNA Kit (TIANGEN, Beijing, China). The operation was performed according to the manufacturer’s protocol. Subsequently, HPV DNA was amplified with the L1 consensus HPV PGMY09/PGMY11 primer set as described previously [18]. PCR was performed with a 25 ul reaction system, which contained 1 ul (89 ng) DNA template and 0.75 ul DNA Taq polymerase. Amplification was carried out for 40 cycles in the CFX96 Touch^TM^ Real-Time PCR Detection System (BIO-RAD, USA). HPV genotyping was performed using the HPV GenoArray test kit (Hybribio, Chaozhou, China), which identifies 15 high-risk HPV types (HPV type 16, 18, 31, 33, 35, 39, 45, 51, 52, 53, 56, 58, 59, 66, and 68) and 6 low-risk HPV types (HPV type 6, 11, 42, 43, 44, and 81) using flow-through hybridization and gene chips. The result of genotyping indicated that this patient was not infected with HPV (Fig. 3).

## Discussion

Clear cell/signet-ring cell morphology is no longer restricted to adenocarcinoma. A set of malignant or benign squamous cell lesions has been demonstrated to exhibit clear cell/signet-ring cell morphology. According to our knowledge, this is the first report of the clear cell/signet-ring cell variant of cSCC in the right thigh of a patient with DM. In the cytoplasm of clear cells/signet-ring cells, the septa of vacuoles displayed positivity for PAS and cytokeratin may be composed of intermediate filaments. Electron microscopy revealed that most of the vacuoles contained moderate amounts of electron-dense flocculent material, while the others are empty [19]. Further studies demonstrate that inciting factors may promote the formation of dilated endoplasmic reticulum, and that dilated endoplasmic reticula are among the most important components of these vacuoles [20].

The etiology of the clear cell/signet-ring cell variant of cSCC remains largely unknown. The role of UV radiation in the carcinogenesis of cutaneous tumors has long been understood and reported in the literature. Chronic exposure to UV can cause cellular DNA mutations, such as the various *p53* mutations, and the accumulation of genetic abnormalities eventually leads to the formation of tumors [21]. Previous studies showed that clear cell cSCC is spatially distributed mainly in the head and neck (Table 2) [2, 20, 22–27]. However, the sun-exposed time of the thigh in this case was limited, raising the possibility that other molecular mechanisms may contribute to the pathogenesis of clear cell/signet-ring cSCC.

**Table 2:** Summary of cutaneous squamous cell lesions with clear cell/signet-ring cell morphology

Case	Age/Sex	Pathologic Diagnosis	Location	HC		IHC		Etiology	EM	Reference
				positive	negative	positive	negative			
1	69/M	SRSCC	forehead	N/A	Kreyberg Stain	keratin	N/A	N/A	N/A	11
2	50/M	SRSCC	neck	PAS (septum between vacuoles)	Mucicarmine, PAS (vacuoles)	AE1/AE3, MAK 6 Ker, Ker 903, CAM 5.2, CEA (weak), EMA (weak), Ki-67	Leu M1, S-100, HMB-45, Actin, Vimentin, SMA	Not done	Rough ER cisternal dilatation	9
3	79/F	SCC	right cheek	No	colloical iron, PAS	cytokeratin	No	not done	no stained material	12
4	82/M	SCC	left temple	No	colloical iron, PAS	cytokeratin	No	Not done	no stained material	
5	83/M	SCC	right ear	No	colloical iron, PAS	cytokeratin	No	Not done	no stained material	
6	80/M	SCC	forehead	No	colloical iron, PAS	cytokeratin	No	Not done	no stained material	
7	87/M	SCC	frontal scalp	No	colloical iron, PAS	cytokeratin	No	Not done	no stained material	
8	76/M	SCC	forehead	No	colloical iron, PAS	cytokeratin	No	Not done	no stained material	
9	84/F	SRSCC	upper lip	No	mucicarmine, PAS	keratin, P63, EMA	No	Not done	Not done	13
10	66/F	SCC	sole	AB, colloidal iron,	PAS	AE1/AE3, EMA, CK5/6	CK7, CK20, CEA, BerEP4, S100, Her2, ER, Ki-67	HPV 18	Not done	5
11	67/M	SCC	left lateral canthus	No	AB, mucicarmine, PAS	CK5/6, p63, EMA	CK7, Ck20, CEA	Not done	Not done	2
12	83/M	SRSCC	back of finger	No	Mucicarmine, PAS	No	CK20, CEA, vimentin, HMB45, Melan A, desmin	not done	not done	14
13	62/M	CCSCC	left side of face	No	PAS mucicarmine, AB	AE1/AE3	No	N/A	N/A	15
14	78/F	CC/SRSCC	right thigh	PAS	N/A	AE1/AE3, CK5/6, CK14, CK19, p63, FGFR2	CK7, CK8, CK18, CK20, P16, HER-2, p53, Ki-67	No HPV infection	Not done	present study

*HC* histochemistry; *IHC* immunohistochemistry; *EM* electron microscopy; *SCC* squamous cell carcinoma; *SRSCC* signet ring cell squamous cell carcinoma; *CCSCC* clear cell squamous cell carcinoma; *CC/SRSCC* clear cell/signet ring cell squamous cell carcinoma; *PAS* periodic-acid Schiff; *CCS* clear cell sarcoma; *MiTF* microphthalmia transcription factor; *CCSCC* clear cell/signet ring cell squamous cell carcinoma

The particularly strong association between HPV, especially high-risk varieties of HPV, and the carcinogenesis of cSCC has been confirmed [25]. HPV proteins E6 and E7 can promote the malignant transformation of tumor cells through their interaction with the p53 protein and RB-susceptibility gene product (Rb). P53 is a transcriptional factor that plays a critical role in the regulation of the cell cycle, DNA repair, and apoptosis. HPV can enhance the activity of the ubiquitin pathway and promote the degradation of p53, this is a common event in the initial stages of many malignant tumors [6].

**Fig. 4:**
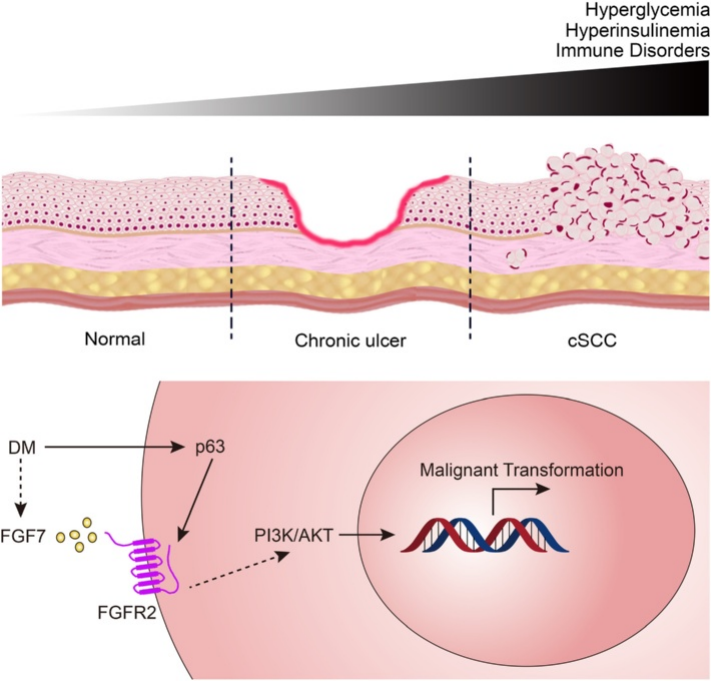
Hypothesis regarding the involvement of type 2 DM in the pathogenesis of cSCC. Epidemiological studies suggest that hyperglycemia, hyperinsulinemia, and immune disorders in patients with type 2 DM increase the risk of malignant transformation. DM-related chronic ulcer is proposed as a precancerous lesion. Type 2 DM can enhance the expression of p63, which in turn activate the downstream element FGFR2. It may also increase the secretion of FGF7 in stromal cells and affect FGFR2 through paracrine stimulates. Indirect evidence indicates that PI3K/AKT signaling may be involved and contribute to the malignant transformation of squamous cell

Although p53 expression was not observed in malignant clear cells/signet-ring cells in this case, we did not detect any HPV infection in this patient, which suggests that it is possible that different etiologic mechanisms exist. However, immunohistochemistry in this case confirmed the up-regulation of p63, another member of the p53 family contributing to the proliferative potential of epidermal progenitor cells [28]. TAp63 isoforms have an effect on the commitment to stratification, while ΔNp63 isoforms regulate the epidermal morphogenesis at a later stage [29]. ΔNp63 not only directly competes with other members of the p53 family in the inhibition of consequent signaling pathways, but also regulates the transcription of several genes involved in tumoreigenesis, such as IRF6, IKKα and FGFR2 (Fig. 4) [30–32].

Accumulating evidence supports the hypothesis that DM, in addition to UV radiation and HPV infection, may be involved in the carcinogenesis of cSCC. Patients with DM have an increased susceptibility to infections and typically exhibit chronic ulcers in the lower extremities. Cases of cutaneous SCC arising in long-standing foot ulcer of diabetic patients have been reported [12–15].

In this study, the clear cell/signet-ring cell variant of cSCC also exhibited a close spatial and temporal relationship with adjacent scars caused by a chronic ulcer. Substantial research further demonstrates that DM patients with oral SCC at advanced stages tended to have a lower overall survival and a higher recurrence rate compared to nondiabetic patients [16]. Epidemiological studies consistently produce results that metformin, a first-line drug for type 2 DM, can significantly reduce the risk of many cancers. Metformin monotherapy is associated with lower risk of breast and gastrointestinal cancers compared to treatment with sulfonylurea (SU) derivatives, insulin or a combined SU and metformin treatment [33, 34]. It can also significantly improve the overall survival rate of patients with head and neck cSCC [17].

Several hypotheses emerge regarding the underlying molecular mechanisms. One study suggested that FGFR2, a downstream effector of p63, may contribute to the progression from squamous cell dysplasia to cSCC [35]. Our study supports this view since clear cells/signet-ring cells exhibited strong-diffuse positivity for FGFR2 (Fig. 4). It is worth noting that, in contrast to our findings, an elaborate study took advantage of an established type 1 diabetic rat model to demonstrate that up-regulated erb-B2 and erb-B3 promoted cell proliferation and inhibited apoptosis [36]. However, species, type of diabetes, and exact cSCC region may explain these different conclusions.

## Conclusion

cSCC tumors with clear cell/signet ring cell morphology are extremely rare, and occur mainly in sun-exposed regions of the body such as the head and neck. We reported our analysis of the first known case of clear cell/signet ring cell cSCC in the right thigh of a woman with type 2 DM. Results of PAS and cytokeratin staining suggested that the septa of cytoplasmic vacuoles in cancer cells were composed of intermediate filaments. The negative result of the HPV type-specific PCR and genotyping indicated that HPV did not affect the malignant transformation of the lesion in this case. Although we have provided preliminary evidence that DM may be involved in the development of clear cell/signet ring cell cSCC via the p63/FGFR2 axis, further research will be required to determine the exact molecular mechanisms involved.

### Consent

Written informed consent was obtained from the patient for publication of this Case Report and any accompanying images. A copy of the written consent is available for review by the Editor-in-Chief of this journal.

## Abbreviations

AB: alcian blue
cSSC: cutaneous squamous cell carcinoma
DM: diabetes mellitus
FGFR2: fibroblast growth factor receptor-2
HPV: human papillomavirus
OTR: organ transplant recipients
PAS: periodic acid Schiff
SCC: squamous cell carcinoma
SU: sulfonylurea
UV: ultraviolet
